# *AEducaAR*, Anatomical Education in Augmented Reality: A Pilot Experience of an Innovative Educational Tool Combining AR Technology and 3D Printing

**DOI:** 10.3390/ijerph19031024

**Published:** 2022-01-18

**Authors:** Laura Cercenelli, Alessia De Stefano, Anna Maria Billi, Alessandra Ruggeri, Emanuela Marcelli, Claudio Marchetti, Lucia Manzoli, Stefano Ratti, Giovanni Badiali

**Affiliations:** 1 eDIMES Lab-Laboratory of Bioengineering, Department of Experimental Diagnostic and Specialty Medicine, University of Bologna, 40138 Bologna, Italy; laura.cercenelli@unibo.it (L.C.); emanuela.marcelli@unibo.it (E.M.); 2 Cellular Signalling Laboratory, Department of Biomedical and Neuromotor Sciences (DIBINEM), University of Bologna, 40126 Bologna, Italy; alessia.destefano3@unibo.it (A.D.S.); annamaria.billi@unibo.it (A.M.B.); alessandra.ruggeri@unibo.it (A.R.); lucia.manzoli@unibo.it (L.M.); 3 Department of Biomedical and Neuromotor Sciences (DIBINEM), University of Bologna, 40126 Bologna, Italy; claudio.marchetti@unibo.it (C.M.); giovanni.badiali@unibo.it (G.B.); 4 Department of Maxillo-Facial Surgery, IRCCS Azienda Ospedaliero-Universitaria di Bologna, 40138 Bologna, Italy

**Keywords:** augmented reality, 3D printing, human anatomy, medical training, medical technologies, medical students, learning approach

## Abstract

Gross anatomy knowledge is an essential element for medical students in their education, and nowadays, cadaver-based instruction represents the main instructional tool able to provide three-dimensional (3D) and topographical comprehensions. The aim of the study was to develop and test a prototype of an innovative tool for medical education in human anatomy based on the combination of augmented reality (AR) technology and a tangible 3D printed model that can be explored and manipulated by trainees, thus favoring a three-dimensional and topographical learning approach. After development of the tool, called *AEducaAR* (Anatomical Education with Augmented Reality), it was tested and evaluated by 62 second-year degree medical students attending the human anatomy course at the International School of Medicine and Surgery of the University of Bologna. Students were divided into two groups: *AEducaAR*-based learning (“*AEducaAR* group”) was compared to standard learning using human anatomy atlas (“Control group”). Both groups performed an objective test and an anonymous questionnaire. In the objective test, the results showed no significant difference between the two learning methods; instead, in the questionnaire, students showed enthusiasm and interest for the new tool and highlighted its training potentiality in open-ended comments. Therefore, the presented *AEducaAR* tool, once implemented, may contribute to enhancing students’ motivation for learning, increasing long-term memory retention and 3D comprehension of anatomical structures. Moreover, this new tool might help medical students to approach to innovative medical devices and technologies useful in their future careers.

## 1. Introduction

Gross anatomy is a complex and fundamental element for medical students in their education [1]. Anatomical knowledge supports the examination of a patient, the formation of a diagnosis, and communication of these findings to the patient and other medical professionals [2], although, in Europe, the Bologna Process [3] led to use of the European Credit Transfer and Accumulation System (ECTS) to correlate the volume of learning. Based on the defined learning outcomes and their associated workload, the amount of information that students have to learn increases every year. Therefore, in the last few years, the range of teaching resources and strategies are under investigation with the aim of creating suggestions for the best teaching practices in this area [4]. Until the late twentieth century, primary anatomical sciences education was mainly dependent on the mainstays of printed textbooks, chalkboard, and photographic projection-based classroom lectures [5]. Three-dimensional (3D) and topographical comprehensions are crucial aspects in medical education [6,7] as well as participatory learning [8]. As a result, the development of new teaching strategies should take these aspects into account. Cadaver-based instruction represents a milestone in the study of anatomy and has survived as the main instructional tool able to provide a realistic and participatory learning experience for hundreds of years. However, the increasing number of medical students and the decreasing availability of donors led to the search for innovative teaching strategies [9,10]. Alternative teaching methods include living or surface anatomy, e.g., the use of body painting [11], plastic anatomical models, medical imaging, and e-learning, which encompasses a wide range of tools, such as computer-based atlases and tutorials [12]. Most of these new educational strategies are technology-based. One technology is virtual reality (VR), i.e., the use of computer modeling and simulation that enables a person to interact with a 3D visual or other sensory environments. Over the last few decades, VR allowed students to visualize, dissect, and interact with simulated objects in artificial 3D space: digital anatomical tables are virtual life-size 2D dissection platforms with a multitouch screen that can be used to explore the anatomy of the whole body. Another opportunity is augmented reality (AR). AR technology combines the real world with computer-generated objects that appear to coexist in the same space as the real world. The virtual scene generated by the computer is designed to enhance the user’s sensory perception of the world that they are seeing or interacting with. Unlike VR, which creates a totally artificial environment, AR uses the existing real environment and overlays new information on top of it, thus providing a composite view. In recent years, AR has been proposed and applied as an aiding tool in many healthcare sectors, including neurosurgery [13], urology [14,15,16], orthopedics [17,18], and craniomaxillofacial surgery [19,20,21,22,23]. AR is rapidly growing with many new potential applications also in the medical education field [24]. To date, AR applications have been adapted to every stage of medical training as anatomical teaching tools [25], classroom study aids [26,27], image training simulators [28], and clinical skills interaction simulators [29]. The aim of this study was to develop and test a prototype of an innovative AR-based tool for medical education in human anatomy (*AEducaAR*, i.e., Anatomical Education in Augmented Reality). The uniqueness of the *AEducaAR* tool lies in the combination of virtual information projected in AR with real tangible 3D-printed anatomical parts that can be explored and manipulated by trainees, thus favoring a three-dimensional and topographical learning approach.

## 2. Materials and Methods

### 2.1. AEducaAR Tool Development

The development phase of the *AEducaAR* tool consists of three steps: (1) Image segmentation and virtual content preparation; (2) Design and manufacturing of the human skull phantom and templates for practical tasks after the studying session; (3) Development of the AR application usable via tablet or HoloLens 2 headset.

#### 2.1.1. Image Segmentation and Virtual Content Preparation

**Figure 1:**
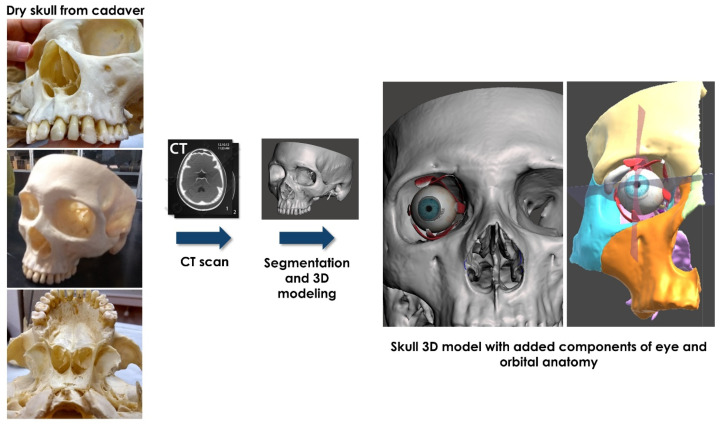
Reconstructed virtual model of dry skull from cadaver CT scan and the added digital content of eye anatomy and facial bones.

The process started from the acquisition of computed tomography (CT) datasets of a dry skull of a human cadaver made available by the Human Anatomy Department of University of Bologna. Bone segmentation was performed using D2P™ software (3D Systems Inc., Rock Hill, SC, USA), and 3D mesh of the dry skull was then generated and saved in STL format. Three-dimensional models of eye anatomy, including eyeball, pupil, orbital muscles, and optical nerve, were selected from Unity asset store (https://assetstore.unity.com/packages/3d/characters/eye-anatomy-animated-100727) (accessed on 20 December 2021). Facial bony structures, lacrimal gland and nerves innervating the orbital muscles were added, starting from real patient datasets and using MeshMixer software (Autodesk Inc., San Rafael, CA, USA) for 3D sculping and mesh smoothing of the anatomical components. All these digital anatomical models were used as virtual content in the *AEducaAR* application. Additional infographic, such as sagittal and transversal planes that divide the orbit into four sectors, was included (Figure 1). 

#### 2.1.2. Manufacturing of Human Skull Phantom and Templates for Practical Tasks

**Figure 2:**
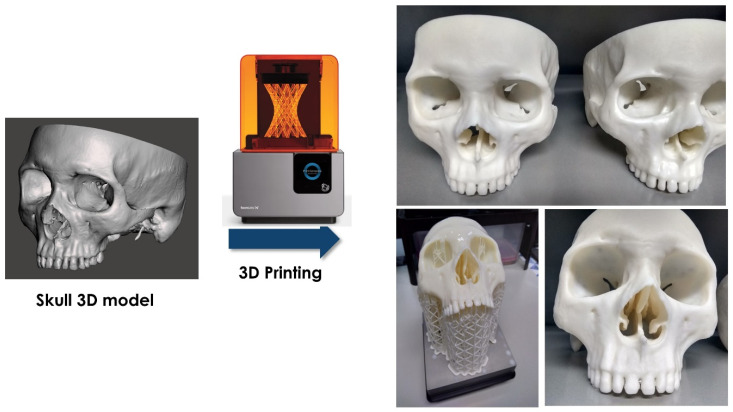
The obtained 3D printed phantom of the human skull.

**Figure 3:**
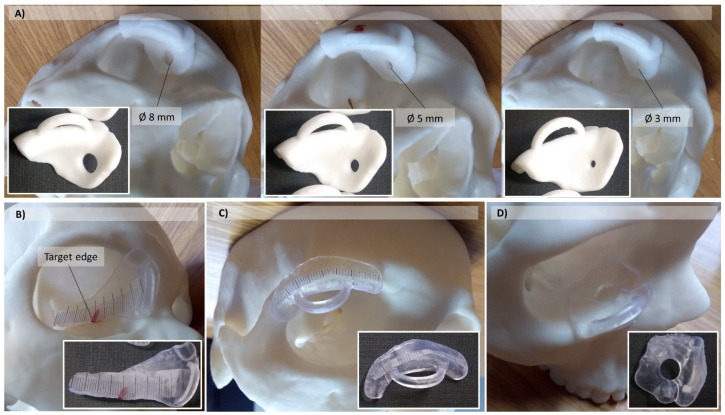
CAD/CAM templates applied to the 3D printed skull model for performance evaluation in practical tasks involving specific anatomical structures: trochlea with an inspection window of different diameters (**A**); posterior edge of the eyeball (**B**); lacrimal gland (**C**); bone insertion of inferior oblique muscle (**D**).

From the skull 3D model of the previous step, a tangible phantom made of photosensitive resin was produced via a stereolithography (SLA) 3D printer (Form 2, Formlabs, Somerville, MA, USA) using a standard white resin (Figure 2). In order to evaluate the students’ performance in executing some practical tasks on the 3D printed skull, after the studying session (see the following sections), four different templates were designed and 3D printed (Form 2, Formlabs, Somerville, MA, USA) to match the surface of the phantom model. One template was produced for each of the anatomical structures involved in the practical tasks (Figure 3): the trochlea (A); the posterior edge of the eyeball (B); the lacrimal gland (C); the bone insertion of the inferior oblique muscle (D). The trochlea template was provided with an inspection circular window of different diameters (3 mm, 5 mm, 8 mm) in order to evaluate various levels of accuracy in performing the task.

#### 2.1.3. Development of AR Application

The obtained virtual models of the skull, eye, and orbital anatomy were imported into Unity 3D software (Unity Technologies, San Francisco, CA, USA) extended with the Vuforia Engine package (PTC, Inc., Boston, MA, USA), which is a specific software development kit for creating augmented reality apps. The tracking algorithm and registration between the virtual content and the real scene were implemented using the model target function of Vuforia Engine, which allows the markless tracking of a physical object by recognition of the shape of the 3D object itself observed from a certain perspective. In this specific case, the skull 3D model obtained from cadaver dry skull was used as the model target for virtual content registration.

**Figure 4:**
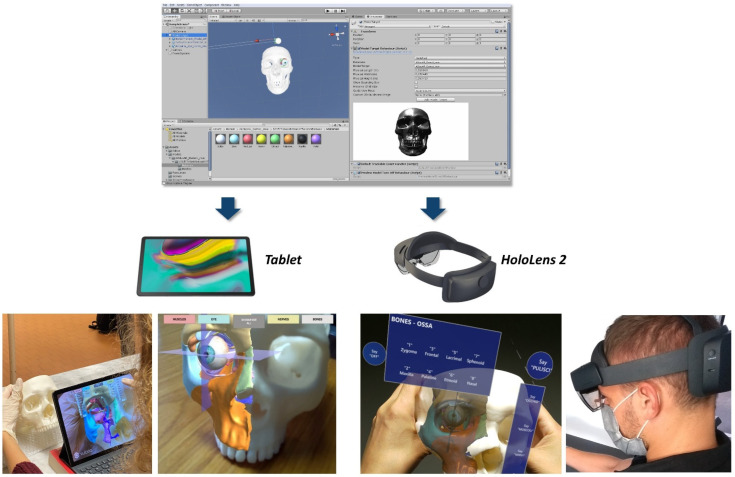
The *AEducaAR* application implemented for use on tablet or on HoloLens 2 smart glasses.

The created AR application was built as an Android app for mobile devices, in order to be used on a tablet device (Samsung Galaxy TAB S5E) (Figure 4). 

Interactive user interface toggles (check box) and panels were added to turn off and on the rendering of each virtual anatomical structure (eye, muscles, nerves, bones).

The application was also built as a Universal Windows Platform (UWP) app for AR glasses in order to be used on Microsoft HoloLens 2 headset. In this case, voice commands to show/hide virtual anatomical structures were also implemented to provide a completely hands-free AR system (Figure 4).

### 2.2. Study Design

**Figure 5:**
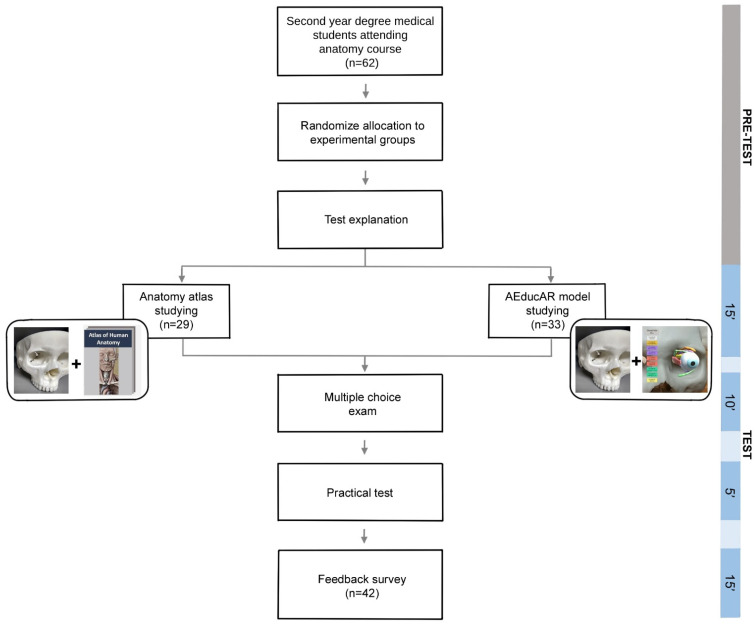
Study design flowchart.

During the first and the second semester of the 2020/2021 academic year, a total of 62 second-year degree medical students attending the human anatomy course at the International School of Medicine and Surgery of the University of Bologna were included in this study. Volunteer participants were recruited during the anatomy class. The students were divided randomly into two groups: one group using the AR application and the 3D printed skull model (*AEducaAR* group), and one control group (CNTRL group) using the human anatomy atlas and the same 3D printed skull. The aim was to compare the two different learning approaches (Figure 5).

Thirty-three participants were included in the *AEducaAR* group, and 29 in the CNTRL group. Before starting the trial, the CNTRL group was informed that they could also gain experience with the *AEducaAR* tool after the test session. This option was given to the CNTRL group in order to not alter the emotional effect induced by possibly perceiving exclusion from trying new technologies. For this first pilot trial of the *AEducaAR* tool, the anatomical region of the orbit was chosen. The reasons behind this decision included starting from a region including many structures (bones, muscles, and nerves), and where the topographical relationships between anatomical structures in the three-dimensional space are quite difficult. The two groups spent 15 min studying the anatomical region of the orbit. The *AEducaAR* group studied using the *AEducaAR* tool (AR application on tablet + 3D printed skull), while the CNTRL group used a classical approach (atlas + 3D printed skull). All the students attended a class on the anatomical region of the orbit one week before the test without knowing what the topic of the test would be. The measurement of the achieved learning in each group was performed using an objective test (multiple-choice exam to be delivered in ten minutes) with ten as a maximum score, and with a practical task where the students were asked to mark the exact position of different anatomical structures on the 3D printed skull. More specifically, the structures were the trochlea, the posterior edge of the eyeball, the lacrimal gland, and the bone insertion of the inferior oblique muscle. The multiple-choice test and the practical tasks were designed by the authors in order to test both theoretical and practical knowledge. Finally, at the end of these activities, an anonymous “feedback” survey was administered to participants from both groups to assess the students’ perception of the *AEducaAR* model. The questionnaire (see Supplementary Table S1) contained four open-ended questions and six Likert scale six-itemed questions.

### 2.3. Statistics

Multiple-choice exam results were reported as mean values and standard deviation (SD). *t*-test for unpaired data was used to compare the mean learning results achieved by the two groups. Graph Pad Prism 5.0 software (San Diego, CA, USA) was used to perform the statistical analysis, and a *p*-value of <0.05 was considered statistically significant.

## 3. Results

### 3.1. The Resulting AEducaAR Tool

The pilot *AEducaAR* tool for studying the orbital anatomy was successfully implemented both as a tablet-based solution (see Supplementary Material Video S1) and a HoloLens-based solution (see Supplementary Material Video S2). Skull tracking and registration between the real and the virtual content resulted as quite sensitive to ambient light conditions in both solutions. The brightness of the virtual content was lower in the HoloLens display than in the tablet. The AR tool on the tablet seems to guarantee a more robust tracking when changing the user viewpoint with respect to the tracked 3D printed skull, while the HoloLens app exhibited a noticeable lag when the skull is manipulated by the student. However, the HoloLens solution provides greater usability and ergonomics, and it allows the student to freely manipulate the 3D printed skull.

### 3.2. Test Results

**Figure 6:**
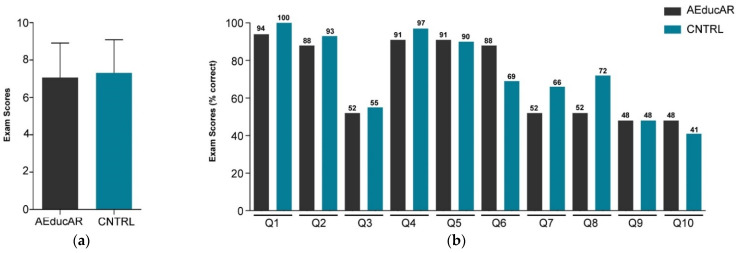
Multiple-choice exam results: (**a**) Mean scores from multiple-choice exam in *AEducaAR* group (*n* = 33) and Control (CNTRL) group (*n* = 29); error bars indicate the standard deviations. (**b**) Scores from multiple-choice exam divided into questions (Q1–10) and displayed as mean percentage of points earned (% correct).

**Figure 7:**
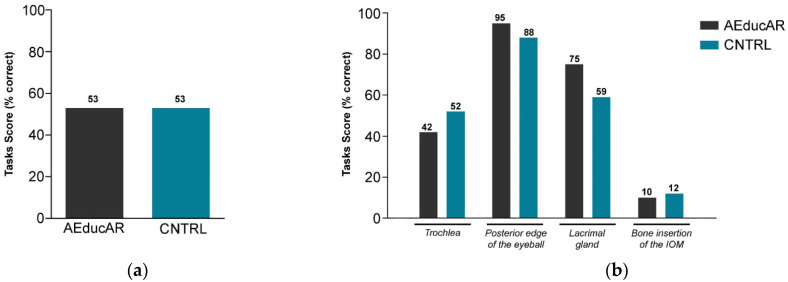
Practical tasks results: (**a**) Overall scores from practical tasks in *AEducaAR* group (*n* = 33) and Control (CNTRL) group (*n* = 29), displayed as mean percentage of points earned (% correct). (**b**) Scores from practical tasks divided into the four anatomical structures involved, displayed as mean percentage of points earned. IOM = inferior oblique muscle.

Comparing the results of the multiple-choice exam administrated after the studying session, the two groups showed a very similar number of correct answers on average, without any statistical significance (Figure 6a). In addition, the distribution among the ten questions was similar (Figure 6b). Comparing the results for practical tasks performed after the studying session, the overall percentage of anatomical structures which was correctly marked on the 3D printed skull was 53% in both groups (Figure 7a). The analysis of each task shows slight differences: the trochlea was correctly located in 42% of the *AEducaAR* group and in 52% of the CNTRL group; the posterior edge of the eyeball was correctly located in 95% of the *AEducaAR* group and in 88% of CNTRL group; the lacrimal gland was correctly located in 75% of *AEducaAR* group and 59% of CNTRL group; the bone insertion of the inferior oblique muscle was correctly located in 10% of *AEducaAR* group and 12% of CNTRL group (Figure 7b). Overall, these results do not highlight significant differences between the *AEducaAR* and CNTRL groups.

### 3.3. Survey Results

This section contains a detailed analysis of the results we obtained from the anonymous “feedback” survey. Forty-three of the 62 students, from both groups, participated by filling out the anonymous questionnaire. The CNTRL group spent 15 min experiencing the *AEducaAR* device after the multiple-choice exam and practical tasks, then they filled the questionnaire. 

**Figure 8:**
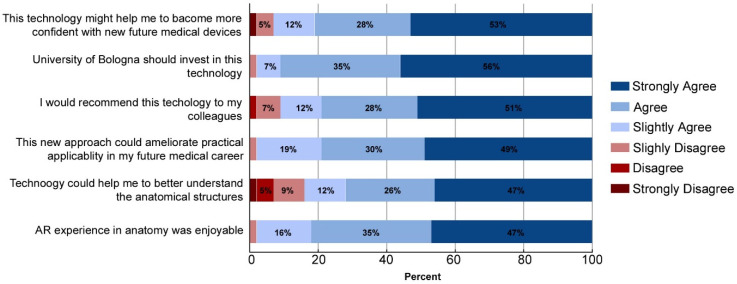
Anonymous questionnaire six-item Likert scale: Students perception on *AEducaAR* device in the CNTRL and *AEducaAR* groups combined (*n* = 42). Different colors in bars represent each different six-item Likert scale option (0 = strongly disagree, 1 = disagree, 2 = slightly disagree, 3 = slightly agree, 4 = agree, 5 = strongly agree). Before taking the anonymous questionnaire, the CNTRL group experienced the AR device.

Figure 8 encapsulates the results from the six explicit statements which were answered using a six-item Likert scale. Overall, the students evaluated the *AEducaAR* tool positively. From the participants, 47% of students strongly agreed and 35% agreed that it was as an enjoyable experience. Compared to anatomy atlas studying, the AR tool was considered beneficial by most students (47% strongly agreed and 26% agreed) to carefully understand anatomy structures, to improve learning, and to be helpful in future medical practice (49% strongly agreed and 30% agreed). Moreover, the largest percentage of students strongly agreed (51%) that the *AEducaAR* tool might help them to become more confident with new future medical technologies. Overall, 51% of students would recommend this technology to their colleagues, and 56% thought that the University of Bologna should invest further in this technology. Students’ open-ended responses were thematically organized in Table 1. Positive responses on considering the experience enjoyable referred to the possibility to test a new learning method. Moreover, students rated the *AEducaAR* tool as more useful and engaging compared with textbook study. 

**Figure 9:**
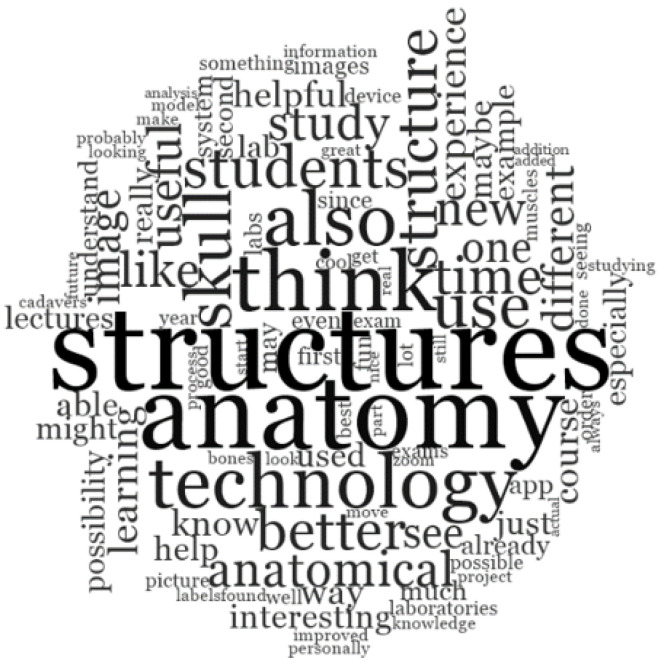
“Word frequency scheme from NVivo software”: scheme of most used words in open-ended considerations. All the words were analyzed by NVivo and each of them were graphed in various dimensions depending on how many times they were repeated in open-ended considerations.

The questionnaire investigated the students’ perception about the best time to use this technology during the course of studies. Responses largely indicate (87%) the second year, which represents the year focused on gross anatomy, as the best period and 13% of students indicated the whole course duration of six years. Suggestions to further improve the efficacy and applicability of this technology were collected, such as the possibility to capture a specific section image, to improve resolution, to zoom in/out the virtual content, and to add labels and quizzes during the AR experience. Finally, in the open consideration and suggestion question, few disadvantages and criticisms were mentioned (Table 1). The most used words in open-ended considerations are graphed in “cloud words” from NVivo, a qualitative data analysis computer software package that helps to organize, analyze, and find insights in unstructured or qualitative data such as open-ended survey responses (Figure 9). The most used words were related to anatomical structure, the technology, and learning approaches, highlighting that the tool merges a new practical and topographical learning method.

### 3.4. HoloLens Experience

**Figure 10:**
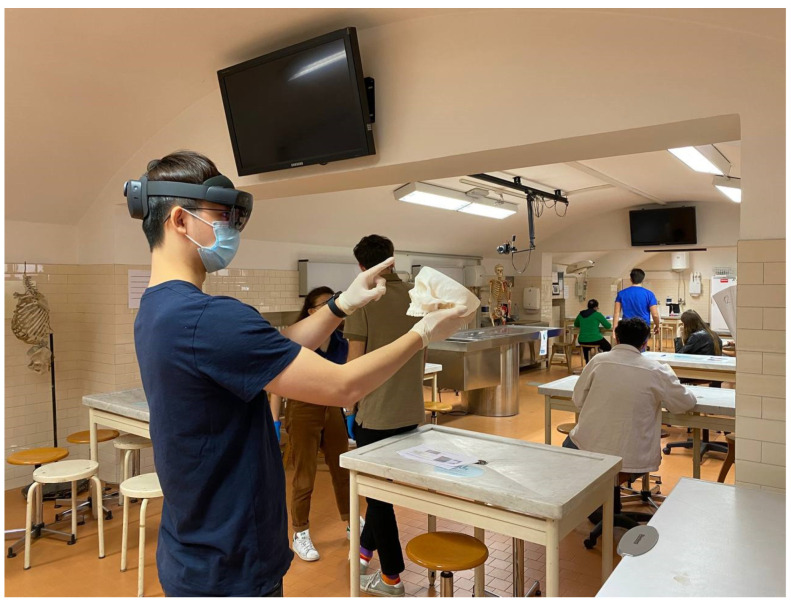
The *AEducaAR* tool experienced with HoloLens 2.

At the end of practical tests, each participant was invited to experience the HoloLens app in order to collect feedback and critical aspects that can be further improved (Figure 10). The students mentioned the experience as “a step in the future”, showing enthusiasm to test it, using terms such as “enjoyable” and “interesting”, such as already mentioned for the *AEducaAR* version displayed on tablet. 

Comments about the HoloLens experience are listed in the last row of Table 1. In the “open consideration and suggestion” category, some criticism referring to HoloLens experience, such as some troubles with software usability and image blurring, can be found. The software usability troubles of the prototype were due to a high learning curve with the students that were not confident with the HoloLens tool. This issue has already been resolved adding a cloned-view screen for the operator in order to be able to visualize what the students were seeing and therefore assisting them in the learning process of the tool itself. Furthermore, problems with image perception were due to the low-lit classroom used for the test.

## 4. Discussion

*AEducaAR* represents a pilot experience of an innovative educational tool for anatomical education that combines the virtual information projected in AR with real tangible 3D-printed anatomical parts. In this study, we described the *AEducaAR* tool development, as well as the learning outcomes and results from a survey administrated to medical students after *AEducaAR* experience. The *AEducaAR* tool was designed and created by a synergic work between anatomists, maxillofacial surgeons, and biomedical engineers at the University of Bologna, aiming to create a new strategy for teaching and learning anatomy. The contribution of various professional figures was a huge value for the project. The expertise of anatomists on content creation helped to find a more useful way to teach human anatomy. The maxillofacial surgeons’ knowledge was pivotal for focusing on future applicability of the tool for also training in surgical tasks, and biomedical engineers were fundamental for the technological development of the tool. Our results from the objective test to evaluate the achieved theoretical learning showed that the acquisition of knowledge was independent of the use of the *AEducaAR* tool or classical anatomy atlas, such as already found in previous works in the literature. Indeed, a large study by Dr. Elizabeth A. Duncan-Vaidya, involving approximately 800 students from Cuyahoga Community College (Ohio) enrolled to test the effectiveness of an augmented reality head-mounted display in learning anatomy, showed no difference between traditional and AR-based learning [30]. Moreover, we also evaluated the topographical skills acquisition by administering practical tasks to be performed on the 3D-printed skull model. Even though the overall scores from practical tasks were essentially the same in both groups, the *AEducaAR* group showed better results for some structures (e.g., the posterior edge of the eyeball and the lacrimal gland) compared to the CNTRL group. The reasons behind these results could be related to the specific anatomical topography of each structure evaluated that could be better understood with the innovative AR-based tool compared to the traditional learning strategy based on the atlas. On the other hand, the prototype limits reported in the survey underline that further technological improvements are required to increase the tool’s effectiveness. The pandemic situation and the limited number of *AEducaAR* prototypes did not allow for recruitment of a large number of participants for this first pilot experience. Currently, we have no information on “long-term learning” effects that could be different for the two groups, due to the motivational aspects that may help to internalize concepts and allow for continued retainment [31]. Several studies focused their attention on participants’ satisfaction to demonstrate that students are convinced that augmented reality can help them to have a better learning outcome [32,33]. In a study conducted at Ludwig-Maximilians-Universität (LMU) in Munich, the students rated their motivation, 3D understanding, and advantages of augmented reality over textbooks, and their results were largely positive [32]. As a result, we also focused on students’ reported experiences. The results of the survey clearly confirm that students appreciate *AEducaAR* as an enjoyable experience (82% agree or strongly agree) and would recommend it to their colleagues (79% agree or strongly agree). Further, 93% of students recognized this technology as useful to become more confident with new future medical devices. Indeed, studies carried out in recent years have shown a positive association between video game experience and robotic skills simulators, so the use of new digital technologies for educational purposes seems to have important implications for the evolution of robotic surgery training [34]. In addition, students recognized that the possibility of interacting with the 3D printed skull, of moving it and showing/hiding structures overlaid to it, helps them to better understand the anatomical structures, their localization in the three-dimensional space, and the anatomical topographic relations. These feedbacks were beneficial in determining how the choice of the orbital structure was farsighted, and to focus on the importance of 3D interaction between the learning tool and students’ own bodies [35]. 

Generally, students mentioned more advantages in the use of the *AEducaAR* tool in their open-ended answers, compared with the use of textbooks. Although the atlas has a lot of images for each anatomical structure in different planes, it offers no dynamic way to visualize them; therefore, imagination becomes fundamental for a complete understanding. Instead, the *AEducaAR* application provides a 3D rendering of the anatomical structures and allows students to explore and experience the three-dimensionality of the structures. Participants were also asked what could be upgraded in this technology in order to improve its efficacy and applicability to enhance the project promptly. Feedback was useful to improve the pilot tool and make it a collaborative model between creators and students. Some specific suggestions mentioned were to capture a specific section image, to introduce the zoom in/zoom out of the virtual elements, and to add labels and quizzes during the AR experience. These suggestions could increase the user–system interaction that could make *AEducaAR* more than just a “see-through” tool, and something to actively interact with. Furthermore, the head-mounted solution via the HoloLens 2 smart glasses was evaluated positively in spite of some technical issues that still remain. 

It is also relevant to underline that the forced quarantines due to the COVID-19 pandemic increased the rates of anxiety, depression, and fatigue, especially in students, who are often already affected by these emotions. The abrupt transition to e-learning aroused negative moods in students, affecting long-term learning and retention of concepts. It was reported that anxiety and depression lead to reduced performance and a decrease or absence of motivation [36]. For this reason, the development of new educational strategies, such as *AEducaAR*, may contribute to increasing students’ motivation with the use of new technologies and uncommon tools. The development of learning models that do not necessarily require dedicated or expensive display interfaces could facilitate autonomous learning at home by increasing motivation and decreasing stress. These findings were also concluded in Javier Ferrer-Torregrosa et al.’s 2016 study that compared the use of augmented reality, videos, and notes for autonomous study. Their results showed that AR helped students to keep their attention and it was related to longer study time and fewer distractions than other learning tools. The increased amount of time spent studying was due to greater motivation and a more enjoyable study experience, not to a more difficult understanding [37]. On this basis, the proposed *AEducaAR* tool offers a collaborative educational approach that can change the students’ learning experience in a stimulating way.

## 5. Conclusions

Overall, the results of this pilot study suggest that the *AEducaAR* collaborative model may be a valid educational tool that currently provides an anatomical knowledge acquisition comparable to traditional textbooks. However, the positive student perceptions, almost unanimously, suggest that they would benefit from the use of *AEducaAR* tool. Further improvement of the tool is expected to enhance its learning efficiency, as potentialities of AR and 3D virtual modeling in medical education are expanding. Especially, AR might enhance an “interaction-based” learning approach through the use of interactive tools, such as Mixed Reality Toolkit in HoloLens 2, that allow the user to interact with the projected 3D holograms. Considering all the possible future perspectives in this field of investigation, it will be essential to continue the development and improvement of the *AEducaAR* prototype, increasing its functions and the number of testers who may experience it.

## Figures and Tables

**Table 1:** Open-ended students’ feedback about *AEducaAR* tool (*n* = 43).

Questions	Type of Answers	Number of Answers
Why was *AEducaAR* an enjoyable experience?	Test new learning method	17
	More interesting system compared with textbook study	16
	Useful to see tridimensional structures	8
	No response	2
When do you think it might be the best time to use this technology during your course of studies?	During the second year	20
	During the whole course	17
	No response	6
What could be upgraded in this technology in order to improve its efficacy and applicability?	Capture a specific section image	13
	Improving resolution	8
	Possibility to zoom in/zoom out	5
	Add label	4
	Add quizzes	4
	No response	9
Open consideration and suggestion	Troubles with software	10
	Blurry images	7
	No response	26
Comments about HoloLens experience	More interesting system compared with textbook study	23
	It was enjoyable	12
	It was a step in the future	8

## Data Availability

All data generated or analyzed during this study are included in this published article.

## Supplementary Materials

The following supporting information can be downloaded at: https://www.mdpi.com/article/10.3390/ijerph19031024/s1, Table S1: Anonymous questionnaire provided to the participants; Video S1: *AEducaAR* tool tablet-based solution; Video S2: *AEducaAR* tool HoloLens-based solution.
