# Supplementary material for: AEducaAR, Anatomical Education in Augmented Reality: A Pilot Experience of an Innovative Educational Tool Combining AR Technology and 3D Printing
Source: Int J Environ Res Public Health. 2022 Jan 18;19(3):1024. doi: 10.3390/ijerph19031024 (PMC8834017; doi:10.3390/ijerph19031024)
Supplement: Supplementary file 1 [file ijerph-19-01024-s001.zip › Supplementary Table S1.pdf]

Table S1. AEducAR Experience Feedback.

	Strongly Disagree	Disagree	Slightly Disagree	Slightly Agree	Agree	Strongly Agree
AR experience in anatomy was enjoyable						
Technology could help me to better understand the anatomical structures						
This new approach could ameliorate practical applicability in my future medical career						
I would recommend this technology to my colleagues						
University of Bologna should invest in this technology						
This technology might help me to become more confident with the new future medical devices						

Why was AEducAR an enjoyable experience?<br>[...]
When do you think it might be the best time to use this technology during your course of studies?<br>[…]
What could be upgraded in this technology in order to improve its efficacy and applicability?<br>[…]
Open consideration and suggestion<br>[…]
